# Transcriptional profiling clarifies a program of enzalutamide extreme non-response in lethal prostate cancer

**DOI:** 10.1038/s41698-025-01002-8

**Published:** 2025-07-07

**Authors:** Anbarasu Kumaraswamy, Ya-Mei Hu, Joel A. Yates, Chao Zhang, Eva Rodansky, Dhruv Khokhani, Diana Flores, Zhi Duan, Yi Zhang, Shaadi Tabatabaei, Rachel Slottke, Shangyuan Ye, Primo Lara, Adam Foye, Charles J. Ryan, David A. Quigley, Jiaoti Huang, Rahul Aggarwal, Robert E. Reiter, Max S. Wicha, Tomasz M. Beer, Matthew Rettig, Martin Gleave, Christopher P. Evans, Owen N. Witte, Joshua M. Stuart, George V. Thomas, Felix Y. Feng, Eric J. Small, Zheng Xia, Joshi J. Alumkal

**Affiliations:** 1 Department of Internal Medicine, University of Michigan, Ann Arbor, MI USA; 2 Rogel Cancer Center, University of Michigan, Ann Arbor, MI USA; 3 Department of Biomedical Engineering, Oregon Health & Science University, Portland, OR USA; 4 Knight Cancer Institute, Oregon Health & Science University, Portland, OR USA; 5 Biostatistics Shared Resource, Knight Cancer Institute, Oregon Health & Science University, Portland, OR USA; 6 University of California Davis, Davis, CA USA; 7 Helen Diller Family Comprehensive Cancer Center, University of California San Francisco, San Francisco, CA USA; 8 Department of Medicine, University of California San Francisco, San Francisco, CA USA; 9 Masonic Cancer Center, University of Minnesota, Minneapolis, MN USA; 10 Department of Medicine, Division of Hematology, Oncology and Transplantation, University of Minnesota, Minneapolis, MN USA; 11 Department of Urology, University of California San Francisco, San Francisco, CA USA; 12 Department of Epidemiology & Biostatistics, University of California San Francisco, San Francisco, CA USA; 13 Duke University, Durham, NC USA; 14 Departments of Medicine and Urology, University of California Los Angeles, Los Angeles, CA USA; 15 Department of Medicine, VA Greater Los Angeles Healthcare System, Los Angeles, CA USA; 16 Department of Urological Sciences, University of British Columbia, Vancouver, BC Canada; 17 Vancouver Prostate Centre, University of British Columbia, Vancouver, BC Canada; 18 Department of Microbiology, Immunology, and Molecular Genetics at the David Geffen School of Medicine, University of California Los Angeles, Los Angeles, CA USA; 19 Genomics Institute and Department of Biomolecular Engineering, University of California Santa Cruz, Santa Cruz, CA USA; 20 Departments of Radiation Oncology and Urology, University of California San Francisco, San Francisco, CA USA

**Keywords:** Prostate cancer, Cancer therapeutic resistance

## Abstract

The androgen receptor inhibitor enzalutamide is one of the principal treatments for metastatic prostate cancer. Most patients respond. However, a subset is primary refractory. Seeking to understand enzalutamide extreme non-response (ENR), we analyzed RNA-sequencing in biopsies from men treated prospectively on an enzalutamide clinical trial. We focused on those with ENR (progression within 3 months) vs. long-term response (progression after 24 months). We identified an ENR program linked to proliferation, epithelial-to-mesenchymal transition, and stemness. High expression of this program in additional datasets was independently linked to poor tumor control with AR targeting but favorable tumor control with docetaxel, another standard treatment. CDK2 was implicated in the ENR program. CDK2 suppression reduced the ENR program and viability of ENR program-high prostate cancer models. The ENR gene program is predictive of non-response to AR targeting. Patients whose tumors harbor this program may be good candidates for docetaxel or CDK2 inhibitor clinical trials.

## Introduction

Novel, potent androgen receptor (AR) pathway inhibitors (ARPIs) such as enzalutamide (enza), abiraterone, apalutamide, and darolutamide are commonly used in the treatment of men with prostate cancer. These agents have been shown to improve survival for those with nonmetastatic^1–3^ and metastatic^4–7^ castration-resistant prostate cancer (CRPC). Enza and these other ARPIs have also been shown to increase overall survival (OS) for men with hormone-naïve prostate cancer who are beginning medical castration for the first time^8,9^. However, nearly one-third of patients do not respond to these therapies, and non-responders have a significantly increased risk of death compared to responder patients^4,7–9^.

The clinical trial Genetic and Molecular Mechanisms in Assessing Response in Patients with Prostate Cancer Receiving Enzalutamide Therapy (NCT02099864) sought to characterize tumors from patients with metastatic CRPC who had not previously received enza^10^. We previously reported baseline genomic and transcriptional features in tumors from patients that did or did not exhibit confirmed PSA responses to enza treatment. Tumors from non-responder patients demonstrated significantly lower AR activity as well as upregulation of stemness-associated gene sets^10^.

In this report, we sought to understand the biology of the most aggressive CRPC tumors from that trial that were primary refractory to enza and that progressed rapidly after enza treatment (<3 months) vs. patients who had long-term, durable responses to enza treatment (>24 months). We hypothesized that transcriptional profiling of these two groups of patients would reveal novel insights and potentially nominate treatment strategies for those patients whose disease rapidly progressed with enza therapy. Our results suggest that there are significant differences in the gene expression program between enza extreme non-responders and long-term responders. Our analysis implicates specific transcription factors, kinases, and genes linked to enza primary resistance. Importantly, we identified a gene program of enza extreme non-response. We confirmed this program was linked to shorter tumor control with ARPI treatment in independent patient datasets. Importantly, in contradistinction to ARPI treatment, patients with high expression of this program appear to derive significant benefit with docetaxel chemotherapy—an alternative standard of care treatment for prostate cancer whose use is often delayed until after progression on ARPI treatment. Finally, our analysis implicated the CDK2 kinase as a driver of the ENR program. Using ENR program-high-like cell lines, we confirmed CDK2’s functional importance for promoting survival and activating the ENR program, suggesting a new therapeutic approach to target ENR program-high tumors more effectively.

## Results

### Extreme non-response patients have distinct transcriptional profiles

We sought to compare differences in gene expression between CRPC patients treated with enza whose tumors were least well-controlled vs. those with the most durable control. Using patient survival data from the clinical trial Genetic and Molecular Mechanisms in Assessing Response in Patients with Prostate Cancer Receiving Enzalutamide (NCT02099864)^10^, we classified patients as extreme non-responders (ENRs) (failure to achieve a 50% PSA decline 12 weeks after starting therapy and whose time on treatment was <3 months from starting therapy) vs. long-term responders (LTRs) (achieving a 50% PSA decline 12 weeks after starting therapy and whose time on treatment was >24 months from starting therapy). Among patients enrolled in this trial, four out of 36 (11%) met the definition of ENR, while 14 of 36 (39%) met the definition of LTR. Because our goal was to understand transcriptional differences between the two groups, we focused on patients meeting these criteria for whom RNA-seq data were available (Supplementary Fig. 1, Supplementary Data 1). Thirteen patients (3 ENR and 10 LTR) met these criteria (Supplementary Data 2).

**Fig. 1:**
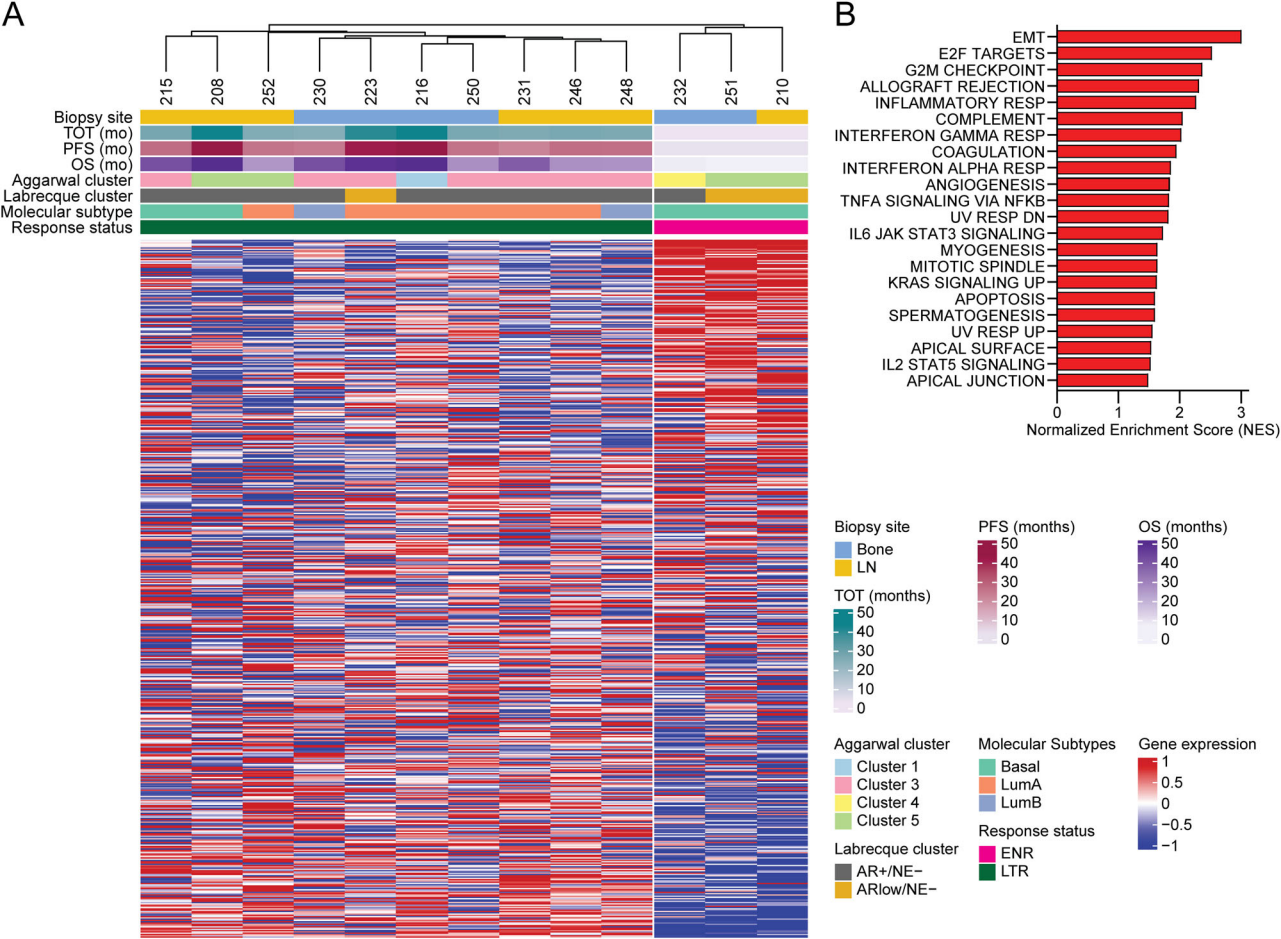
Extreme non-response patients have distinct transcriptional profiles. **A** Heatmap depicting unsupervised clustering analysis separates enza ENR and LTR patients into two distinct transcriptional clusters. Additional classifications such as time on treatment (ToT), progression-free survival (PFS), and overall survival (OS) are shown. Previously published gene signatures^11,12^ are also shown. **B** Gene set enrichment analysis (FDR *q* < 0.05) depicting ENR vs LTR patients.

Using hierarchical clustering, we compared the transcriptional profiles of these thirteen patients, resulting in two clusters that perfectly separated the ENR and LTR groups (Fig. 1A). Additional classifications such as time on treatment (ToT), progression-free survival (PFS), and overall survival (OS) also correlate with extreme non-response. Importantly, several commonly used transcriptional subtyping signatures—including Aggarwal clusters^11^, Labrecque subtypes^12^, and basal/luminal subtyping^13^—were unable to distinguish between the ENR and LTR groups.

To determine pathways that were activated specifically in the ENR patients, we performed gene set enrichment analysis (GSEA) using the Hallmark gene sets (Fig. 1B). Using an FDR q-value cutoff of 0.05, we found 22 significantly upregulated gene sets. Several of these pathways were implicated in our prior report comparing enza non-responders vs. responders based on the failure to achieve a 50% PSA decline 12 weeks after starting enza, including epithelial-to-mesenchymal transition (EMT), IL-6/JAK/STAT3, and inflammatory response^10^. However, the comparison between ENRs and LTRs of focus in this report showed upregulation of several unique pathways related to cell cycle regulation, including E2F targets, G2M checkpoint, and mitotic spindle (Fig. 1B).

**Fig. 2:**
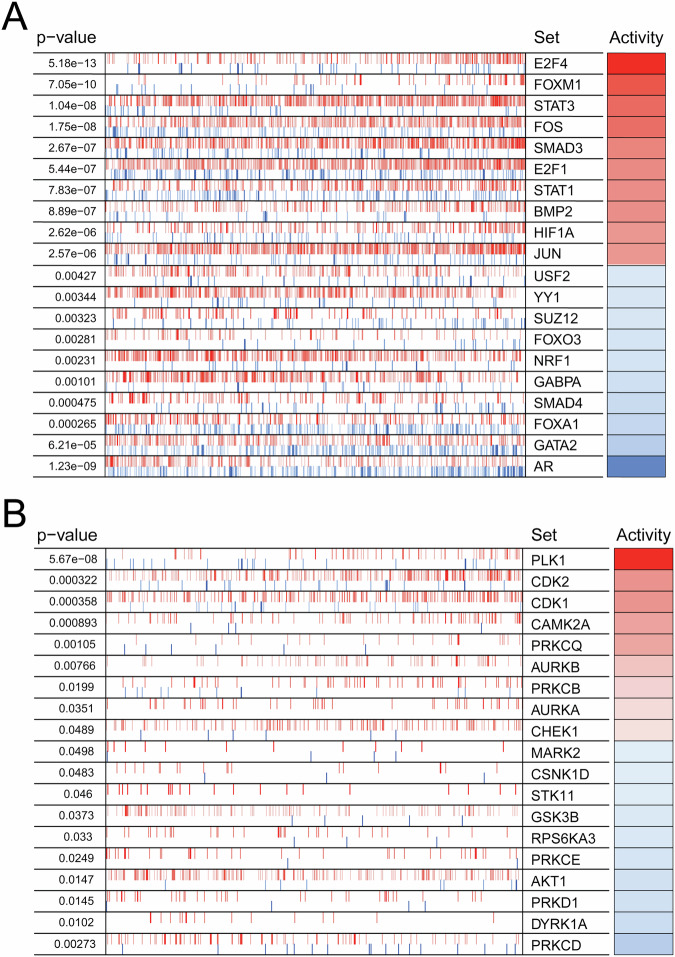
Extreme non-response patients have differential activity of transcription factors and kinases linked to a proliferative program. Master regulator analysis identifies top activated and deactivated transcription factors (**A**) and kinases (**B**) between ENR and LTR patients using the baseline tumor samples. Activity scores (right) and *p*-values (left, calculated using a gene shuffling test of the enrichment scores) were generated using the VIPER R package^14^.

In order to identify differentially activated transcription factors and kinases that are linked to activation of these gene sets, we performed master regulator (MR) analysis^14^. In agreement with the GSEA pathway analysis, several of the most activated transcription factors in ENR patients were involved in cell cycle progression, including E2F4, FOXM1, E2F1, and JUN^15–23^ (Fig. 2A, Supplementary Data 3). Transcription factors with decreased activity in ENR patients included AR and its pioneer factor FOXA1. The most activated kinases included the cell cycle regulators PLK1, CDK2, CDK1, AURKB, and AURKA^24,25^ (Fig. 2B, Supplementary Data 3).

### Identification of an extreme non-response program

**Fig. 3:**
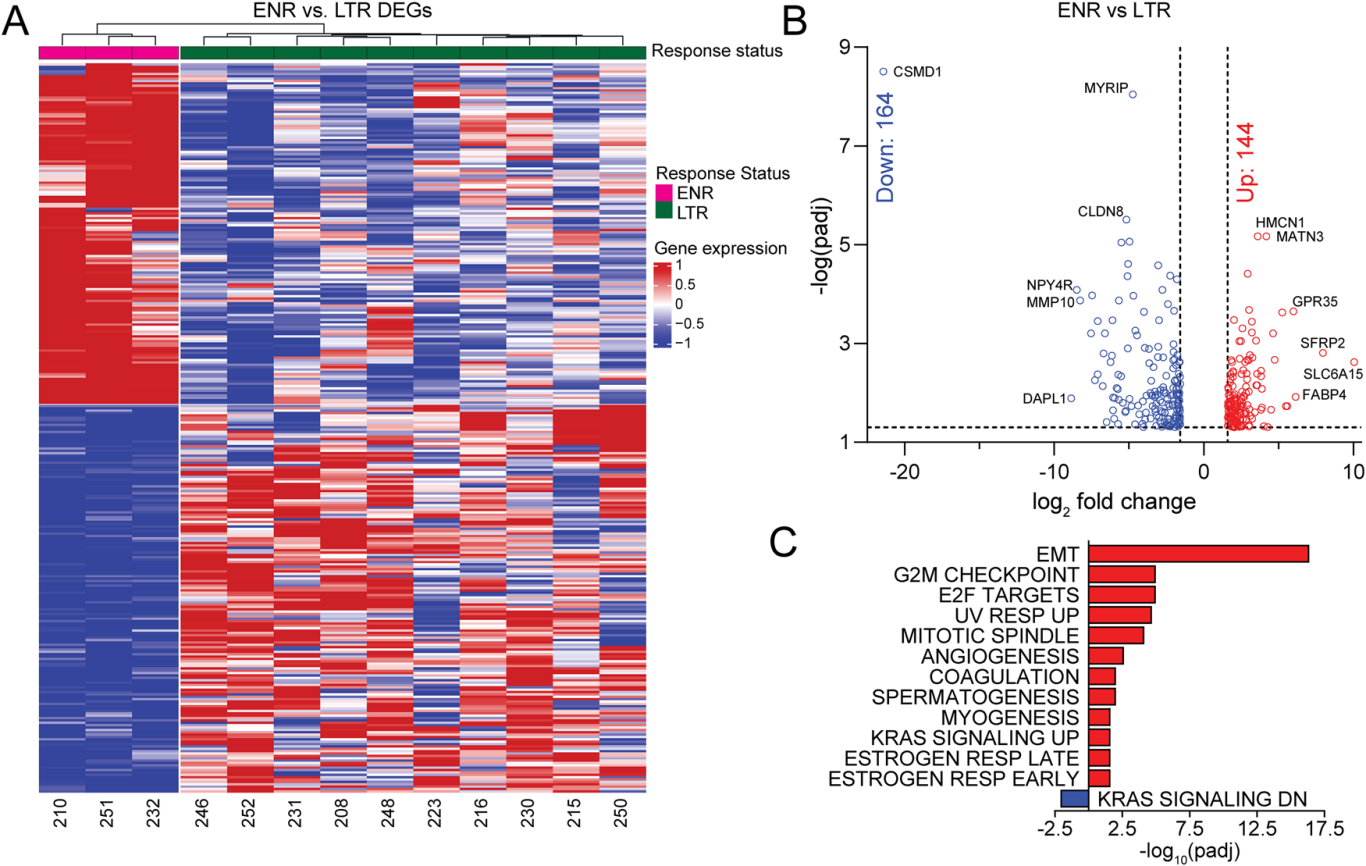
Identification of an extreme non-response program linked to epithelial-mesenchymal transition and proliferation. **A** Heatmap depicting supervised clustering analysis which identified a 308-gene program of extreme non-response. Differentially expressed genes (DEGs) were determined using FC>log_2_(3), padj < 0.05. **B** Volcano plot depicting significant DEGs between ENR and LTR patients is shown. **C** Over-representation analysis of Hallmark pathways represented in the 308 gene extreme non-responder program.

We next sought to identify a gene program enriched in ENR vs. LTR patients. By comparing ENR and LTR transcriptional profiles, we identified a 308-gene program corresponding to extreme non-response to enza treatment (Fig. 3A, Supplementary Data 4). A slight majority (164/308; 53%) of the identified genes were repressed in ENR patient tumors, while nearly half (144/308; 47%) of the genes were activated (Fig. 3B). Using over representation analysis, we determined that pathways relating to a stemness/dedifferentiation program, including EMT, angiogenesis, spermatogenesis, and myogenesis, were among the most activated. Additionally, cell cycle progression pathways, including G2M checkpoint, E2F targets, and mitotic spindle, were also activated (Fig. 3C).

### Extreme non-response program is indicative of a poor response to AR targeted therapy but favorable response to docetaxel chemotherapy

**Fig. 4:**
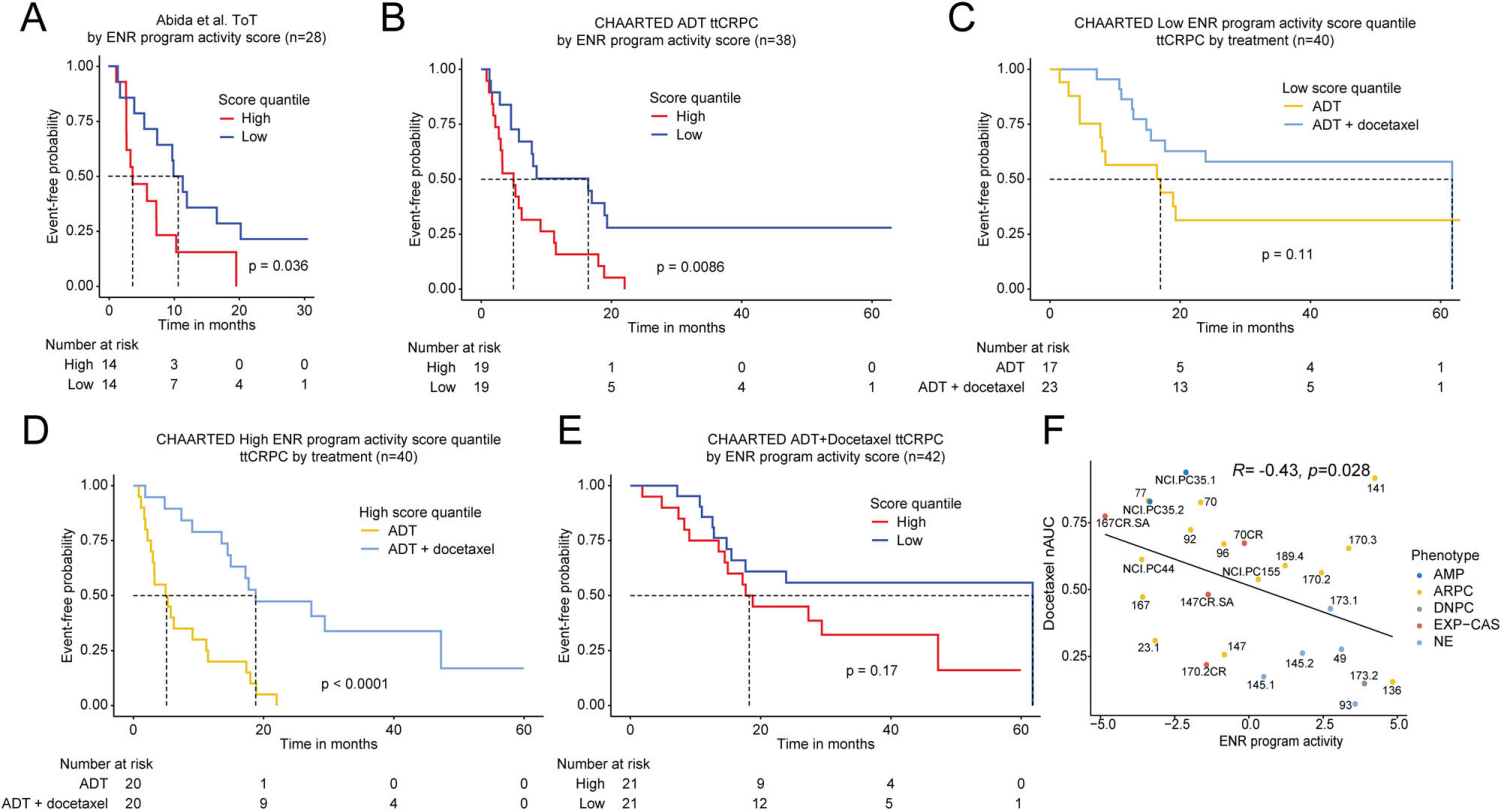
Extreme non-response program is predictive of poor response to AR targeting but favorable response to docetaxel chemotherapy. **A** Kaplan-Meier survival plot of time on treatment (ToT) for patients in the Abida et al.^26^ CRPC cohort stratified by high or low quantile ENR program score. **B** Kaplan-Meier survival plot of time to CRPC (ttCRPC) progression in the CHAARTED^27^ cohort of hormone-naïve prostate cancer patients that received ADT alone stratified by high or low quantile ENR program score. Kaplan-Meier survival plots of ttCRPC progression in the CHAARTED^27^ cohort of hormone-naïve prostate cancer patients in the low (**C**) or high (**D**) quantile of ENR program score stratified by ADT or ADT + docetaxel treatment. **E** Kaplan-Meier survival plot of ttCRPC progression in the CHAARTED^27^ cohort of hormone-naïve prostate cancer patients that received ADT + docetaxel treatment stratified by high or low quantile ENR program score. *p*-values shown were determined using the log-rank test. **F** Correlation analysis of ENR program and sensitivity to docetaxel as indicated by nAUC scores of patient-derived xenograft (PDX) dataset from Senatorov et al.^29^.

We next sought to validate the ENR gene program in independent datasets. We hypothesized that patients with high expression of this program would have less durable response to ARPIs or androgen deprivation therapy (ADT). Using the International Dream Team dataset^26^, we measured the gene program score for evaluable patients with clinical follow-up data. Patients were stratified by quantile, and the high vs. low quantile groups were compared regarding time on first line ARPI—abiraterone or enzalutamide. Patients with a high ENR program score had significantly shorter time on treatment (median = 3.7 months) vs. those patients with a low ENR program score (median = 10.6 months) (Fig. 4A, Supplementary Data 5).

It was unclear whether the ENR program we identified was limited to CRPC patients and only linked to ARPI resistance, rather than being more broadly applicable to patients with hormone-naïve prostate cancer treated with ADT. Therefore, we next examined patients with hormone-naïve prostate cancer from the CHAARTED trial^27^ who were treated with ADT alone or ADT + docetaxel chemotherapy. Importantly, the CHAARTED trial demonstrated that the combination of docetaxel chemotherapy + ADT was superior to ADT alone, but the benefit appeared to be limited to subgroups of patients^28^.

We confirmed that high ENR score was linked to shorter time to CRPC (ttCRPC) in those treated with ADT alone (median = 5.0 months) vs. those with low ENR scores (median = 16.4 months) (Fig. 4B, Supplementary Data 5). Patients with a low ENR score had a similar ttCRPC with ADT or ADT + docetaxel, suggesting these patients do not derive a benefit from adding docetaxel (Fig. 4C). However, those patients with a high ENR program score had a significantly shorter ttCRPC with ADT alone (median = 5.1 months) vs. ADT + docetaxel (median = 18.8 months) (Fig. 4D, Supplementary Data 5). The test for interaction determined there was a strong interaction between high ENR score and docetaxel benefit regarding ttCRPC (*p* = 6.06 × 10^−5^). Among patients that received ADT + docetaxel, those with high ENR scores had no significant difference in ttCRPC vs. those with low ENR scores (Fig. 4E, Supplementary Data 5). Furthermore, the high ENR program remained significant even after performing a multivariable analysis and correcting for covariates known to be linked to shorter ttCRPC (Table 1). To further substantiate our results, we examined expression of the ENR program in a series of prostate cancer patient-derived xenografts (PDXs) for which docetaxel response data were available^29^. The ENR program was highly activated in a subset of PDXs—including several that had an AR-independent, double-negative prostate cancer (DNPC) or neuroendocrine prostate cancer (NEPC) phenotype and several AR-expressing PDXs. The ENR program was strongly associated with docetaxel sensitivity (Fig. 4F). These results strongly suggest the ENR program we identified is predictive of non-response to ARPI or ADT treatment. On the other hand, high ENR program scores are linked to greater tumor control with ADT + docetaxel vs. ADT alone, suggesting this program may be a predictive biomarker for docetaxel response and that docetaxel may be useful for delaying tumor progression in those with high ENR program scores.

**Table 1:** Multivariable analysis demonstrates the independent predictive ability of the extreme non-response program

Dataset	Timepoint	*n*	Variable	Hazard Ratio (95% CI)	*P* value
Alumkal et al.	PFS	20	ENR program	1.21 (1.10–1.43)	<0.0001
			Age	0.93 (0.82–1.05)	0.2609
			ECOG (≥1 vs 0)	4.06 (0.69–24.98)	0.1212
			PSA	0.95 (0.87–1.01)	0.1166
			Gleason score (≥8 vs ≤7)	0.85 (0.22–3.25)	0.8085
Abida et al.	Time on ARSI	41	ENR program	1.08 (1.01–1.17)	0.0329
			Age	0.98 (0.93–1.03)	0.4056
			PSA	1.02 (1.01–1.03)	0.0019
			Gleason score (≥8 vs ≤7)	1.23 (0.60–2.49)	0.5633
CHAARTED	ttCRPC	153	ENR program	1.08 (1.01–1.15)	0.0210
			Age	0.99 (0.97–1.01)	0.2534
			ECOG (≥1 vs 0)	1.60 (1.05–2.40)	0.0294
			PSA	1.00 (1.00–1.00)	0.8576
			Gleason score (≥8 vs ≤7)	1.43 (0.86–2.52)	0.1693
			ADT + docetaxel vs ADT	0.42 (0.29–0.62)	<0.0001
			Disease volume (High vs. Low)	2.39 (1.46–4.14)	0.0004

Multivariable analysis of ENR program correcting for covariates known to be linked to aggressiveness of prostate cancer in patient data from Alumkal et al.^10^, Abida et al.^26^, and CHAARTED^27^ cohorts.

### CDK2 activates the extreme non-response program and promotes survival of extreme non-response program-high prostate cancer cell lines

Seeking to identify other targeting approaches for tumors with a high ENR program, we focused on the kinases implicated in our master regulator analysis since many of these proteins are targetable. PLK1 was predicted to be the top activated kinase. However, clinical trials with PLK1 inhibitors have shown toxicity without significant gains in progression-free survival^30,31^. Therefore, we focused on CDK2, which was the second most highly activated kinase in ENR tumors and for which pharmacologic inhibitors have been developed. Importantly, analysis of gene expression data from International Dream Team dataset^26^, CHAARTED trial^27^, and West Coast Dream Team dataset^32^ revealed a significant positive correlation between CDK2 activity and ENR program score in these patient tumors (Supplementary Fig. 2A).

**Fig. 5:**
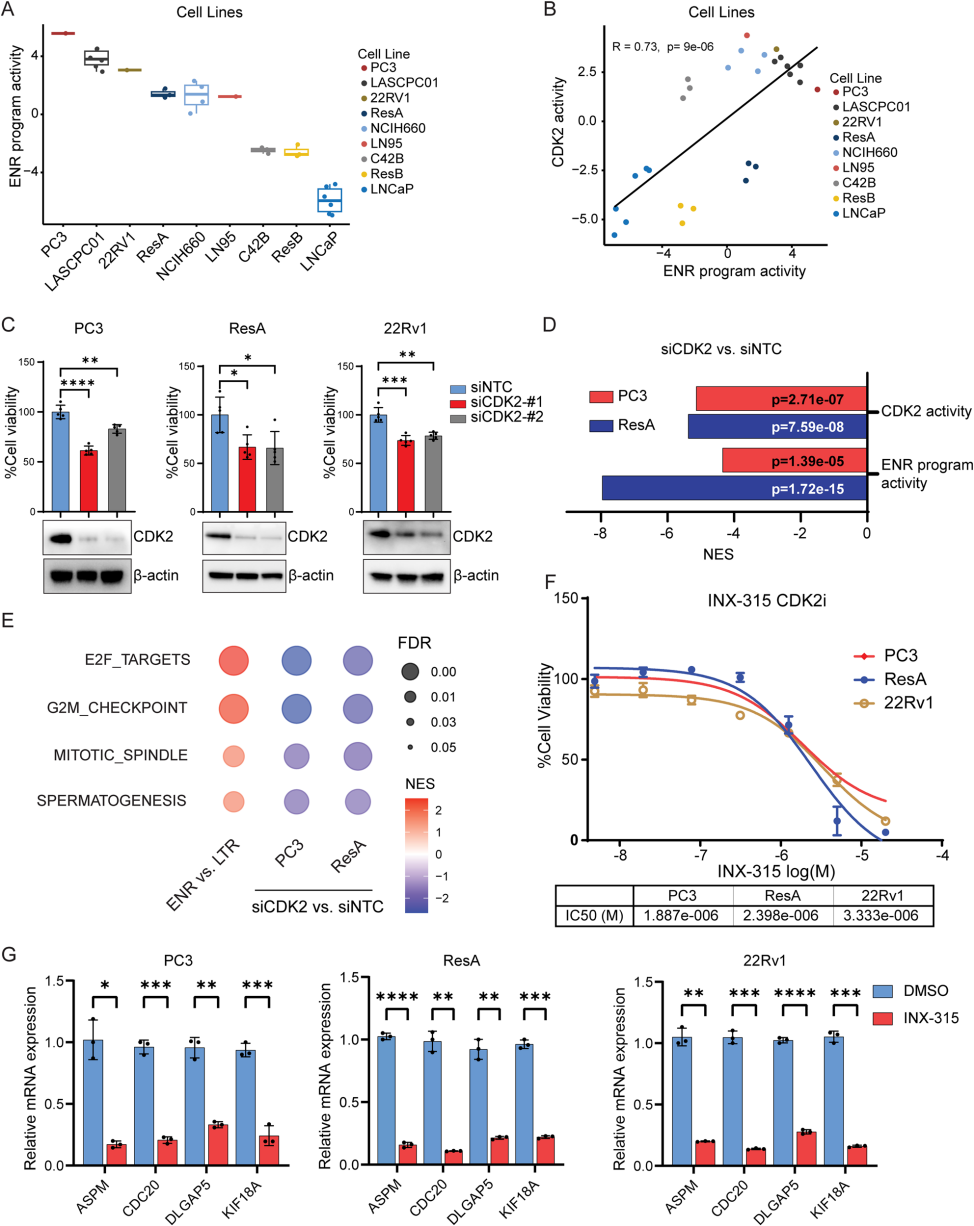
CDK2 activates the extreme non-response program and promotes survival of extreme non-response program-high tumors. **A** Box plot depicting ENR program score in a panel of prostate cancer cell lines analyzed by VIPER. **B** Correlation analysis between ENR program and CDK2 activity score from indicated prostate cancer cell lines analyzed by VIPER. **C** Indicated cell lines were transfected with nontargeting control (siNTC) or two different *CDK2* targeting (siCDK2) siRNAs, and cell viability was measured 96 h after transfection (top). Knockdown of CDK2 was confirmed by Western blot analysis of cell lysates normalized to β-actin (bottom). **D** Bar plot depicting CDK2 or ENR program activity scores from VIPER analysis of RNA-seq from PC3 or ResA cells upon *CDK2* knockdown. **E** Bubble plot depicting pathways from GSEA analysis of RNA-seq data from ENR vs. LTR patients, or upon *CDK2* knockdown in PC3 or ResA cells. **F** Indicated cells were treated with increasing doses of INX-315 for 72 h. IC50 values were calculated from the dose-response viability curves. **G** Indicated cells were treated with DMSO vehicle or 2 µM of INX-315 for 6 h. Expression of ENR program genes were analyzed by qPCR normalized to *β-actin*.

To identify appropriate models to test the functional importance of CDK2, we first examined expression of the 308 gene ENR program in a collection of prostate cancer cell lines. The program was most highly activated in the AR-negative cell line PC3, AR-negative NEPC cell line LASCPC-01, enzalutamide-resistant amphicrine-like 22RV1 cell line that harbors both an AR program and an NEPC program, and an enzalutamide-resistant, AR activity-low cell line called ResA^33^ derived from LNCaP cells after chronic enza treatment (Fig. 5A). Like in patient tumors, there was a strong direct correlation between CDK2 activity and the 308 gene ENR program score in cell lines (Fig. 5B). We also confirmed a positive correlation between ENR program and CDK2 activity in PDXs (Supplementary Fig. 2B), strongly suggesting CDK2 activity may be linked to the ENR program’s activation. Notably, like in the cell lines, many of the ENR program-high, CDK2 activity-high PDXs were AR-null DNPC or NEPC PDXs.

To test the functional importance of CDK2 in ENR program high models, we knocked down *CDK2* using pooled RNAi in three ENR program-high cell lines: PC3, ResA, and 22Rv1. *CDK2* suppression reduced cell viability in each case (Supplementary Fig. 2C). We validated these effects using transfections with two individual siRNA oligos targeting *CDK2* (Fig. 5C). To understand genes regulated by CDK2, we analyzed gene expression changes using RNA-seq upon *CDK2* knockdown in two ENR program-high models—AR-negative PC3 and enzalutamide-resistant, AR activity-low ResA. In each case, *CDK2* knockdown reduced expression of the ENR gene program (Fig. 5D). Moreover, master regulator analysis demonstrated that *CDK2* knockdown suppressed the activity of several TFs (E2F4, E2F1, FOXM1, MYBL2, IRF3) implicated in enza ENR patient tumors (Supplementary Figure 2D). Pathway analysis demonstrated that *CDK2* knockdown led to downregulation of key proliferative pathways such as E2F and cell cycle pathways (Fig. 5E) that were implicated in our analysis of ENR vs. LTR patients (Fig. 1B). Taken together, these data suggest that CDK2 regulates the ENR program and that CDK2 interference is a promising approach to block this program.

Next, we sought to test a pharmacologic approach to blocking CDK2. Therefore, we treated ENR program-high cells with INX-315, that has been shown to suppress CDK2 function in ovarian and breast cancers and that is currently in clinical trials (NCT05735080)^34^. INX-315 treatment recapitulated the effects of *CDK2* RNAi on reducing cell viability (Fig. 5F). INX-315 also reduced expression of ENR program genes that were among the top differentially-expressed genes upon *CDK2* RNAi (Supplementary Data 6) at both early (six hours) and late timepoints (48 h) (Fig. 5G, Supplementary Fig. 3A).

The ENR program is linked to low AR activity (Fig. 2). Indeed, AR-null PC3 and LASCPC-01 had the highest ENR program scores, and these are intrinsically unresponsive to enza due to the absence of AR expression. However, we sought to determine if CDK2 inhibition in acquired enzalutamide resistance, AR activity-low ResA cells, might re-sensitize these cells to enza through re-activation of AR signaling. ResA is normally maintained in enza. Therefore, we first washed ResA cells out of enza for six days and then treated with increasing dose of INX-315 alone or in combination with enza. Compared to cells treated with vehicle, enza did not enhance the growth inhibition observed with INX-315 (Supplementary Fig. 3D). INX-315 did not increase expression of the canonical AR *KLK3* that encodes for the PSA protein, nor did it augment the suppression of *KLK3* observed with enza alone. (Supplementary Fig. 3D). These results suggest INX-315’s effects on the ENR program may primarily contribute to the anti-proliferative effects observed in ResA.

Finally, we sought to determine if the effects of INX-315 were specific to ENR-high models. INX-315 treatment of ENR-low LNCaP cells reduced cell viability (Supplementary Fig. 3C). INX-315 reduced expression of ENR program genes at early timepoints (Supplementary Fig. 3D). However, in contrast with ENR-high models (Supplementary Fig. 3A), INX-315 failed to suppress these same genes at later timepoints (Supplementary Fig. 3E). Prior reports demonstrate CDK2 activates AR function^35–37^. Indeed, INX-315 reduced expression of the canonical AR target, *KLK3*, in LNCaP (Supplementary Fig. 3E). Moreover, combination treatment with INX-315 + enza did not lead to enhanced anti-proliferative effects vs. enza alone (Supplementary Fig. 3F). These results suggest INX-315’s effects on AR signaling may contribute to the anti-proliferative effects of CDK2 inhibition observed in ENR-low LNCaP cells. Altogether, these data suggest that targeting CDK2 may be promising in both ENR-high and ENR-low tumors through modulation of distinct programs.

## Discussion

Resistance to ARPIs remains a major problem leading to morbidity and mortality for prostate cancer patients. While most patients experience transient responses with ARPI treatment, a subset of tumors exhibit de novo resistance and rapid progression, or ENR. Little is known about the biology of ENR tumors. There are no known markers to identify these tumors, and there are no therapies known to improve outcomes for patients with ENR. Our report sheds new light on these unknowns and suggests that ENR tumors harbor an aggressive, proliferative program that may be targetable with existing approved treatments for prostate cancer (i.e., docetaxel). Moreover, our results nominate additional drug targets—CDK2 inhibitors—to target ENR-high tumors.

Unsupervised transcriptional clustering comparing patients with enza ENR to patients with long-term durable responses separated the two groups correctly—which other tumor classification signatures derived on different data^11–13^ were unable to do, indicating that the underlying gene expression program patterns contribute to the biology and pathology of these distinct tumor subsets. GSEA of differentially expressed genes identified 22 pathways (*p* < 0.05) specifically activated in enza ENR patients vs. enza LTR patients. While a majority of these pathways (16/22) overlap with those identified in our previous report linked to general de novo enza resistance and low AR activity^10^, there were several pathways unique to the enza ENR patient cohort. Pathways activated in ENR patients, specifically, were primarily related to cell cycle regulation, including E2F targets, G2M checkpoint, and mitotic spindle. IL2-STAT5 signaling is another pathway activated in enza ENR tumors that has been shown to have a role in castration-resistance, enza resistance, and metastasis^38–40^. Indeed, several studies have identified STAT5 as a potential target in prostate cancer^41–44^. Our results suggest that STAT5 targeting is worthy of further study in overcoming enza ENR.

While unsupervised analysis identified a program of enza ENR, key regulators of this program were not well-known. Master regulator analysis revealed activation of specific transcription factors and kinases—many of which regulate stemness, EMT, and lineage plasticity programs^45,46^. Regarding stemness, OCT1 (POU2F1) activity was increased in ENR patients. OCT1 has been found to promote alternative differentiation programs in AR negative prostate cancer^47,48^. We also observed increased activity of SNAI2 that has been shown to promote EMT and resistance to ADT^49^. Thus, activation of these factors may contribute to the reduced AR activity observed in enza ENR tumors.

Enza ENR tumors also had upregulation of STAT1 and STAT3. STAT3 signaling has been shown to promote enza resistance^50^, and JAK/STAT activation is important for lineage plasticity^51^—a known mechanism of enza resistance^21,46,52,53^. STAT signaling is targetable by JAK inhibition, including with JAK inhibitors that are already approved for use in other diseases^54–56^.

Matching the GSEA pathway analysis, we found increased activity of several proliferative transcription factors including E2F1, E2F4, FOXM1, MYBL2, and JUN in enza ENR patients. Prior work demonstrates that E2F family members are important cooperating partners with the BET bromodomain protein BRD4^21^. A phase Ib trial of the BET bromodomain inhibitor (BETi) ZEN-3694 + enza demonstrated safety, and prolonged responses were seen in patients whose tumors progressed most rapidly with prior abiraterone treatment^57^. Furthermore, in a subset of patients with an NEPC program enrolled on this trial, those with high E2F1 expression or activation of an E2F1-BRD4 program had more durable tumor control^21^. MYBL2 upregulation has also been linked with prostate cancer aggressiveness and resistance to AR targeting and appears to be a key target of the histone demethylase KDM5B^58^.

There are limited biomarkers to identify ARPI-naïve patients with CRPC who will progress rapidly and exhibit ENR. Using supervised clustering and differential gene expression analysis, we identified a 308 gene program of enza ENR. Over representation analysis determined that the most activated gene sets were related to stemness and cell cycle progression, matching results from unsupervised analysis and demonstrating the importance of these pathways for ENR.

Slightly more than half (53%) of the ENR program genes were downregulated vs. LTR tumors. However, KRAS Signaling Down was the only significantly downregulated Hallmark pathway. Prior work demonstrates KRAS or MAPK activation is linked with prostate cancer aggressiveness, and this is explained in part by EZH2-mediated suppression of the negative regulator of Ras^59,60^. Master regulator analysis implicated the AR as the top deactivated transcription factor in enza ENR tumors. In keeping with these results, the AR-associated genes KLK3 and PAK6 were among the downregulated genes in the enza ENR program.

Consistent with an increased stemness program, several genes previously shown to be down regulated in mammary stem cells^61^—ALDH3B2, CLDN8, SORBS2, ENPP4, SGSM3, LYPD3, SORL1, FAAH, ABHD17C, CCT6B, TRIM68, PAOX, CLCN3—and basal cells^62^—ABAT, ALDH3B2, AGR2, PTPRN2, CACNA1D, CAMK2B, RAB27B, SORL1, FAAH, ABCA12, MYT1, TRIM68, CACNA2D2, MAN1C1, GP2, GALNT7—were among the downregulated genes in the enza ENR program. In addition, several genes up regulated in luminal vs mesenchymal breast cancer cells^63^—ABAT, INHBB, ALDH3B2, AGR2, TMEM45B, ENPP5, CACNA1D, LYPD3, RAB27B, RNF144B, SORL1, FAAH, ABCA12, PRSS8, SLC52A3, TMEM134—were downregulated in ENR tumors. Together, the downregulation of these genes may contribute to an EMT phenotype and increased invasive potential.

We validated the ENR gene program using independent datasets of men treated with ARPIs or ADT, respectively. In each case, a high ENR program was linked to shorter tumor control with AR targeting, and there was a strong suggestion this program was not only prognostic but also predictive of failure to respond to AR targeting with enza or other ARPIs such as abiraterone or ADT alone. Importantly, multivariable analysis in all datasets examined demonstrated that a high ENR program was independently associated with worse outcome in the datasets examined.

In examining the CHAARTED dataset of patients with hormone-naïve prostate cancer treated with ADT alone vs. ADT + docetaxel, we found that patients with high ENR program scores did much worse with ADT treatment than patients with low ENR scores. However, high ENR score patients derived significant benefit with ADT + docetaxel vs. ADT alone—a benefit not seen with adding docetaxel in patients with low ENR scores. The test for interaction demonstrated a highly significant interaction between high ENR program score and time to CRPC after docetaxel treatment. In support of our contention that the ENR program is linked to docetaxel response, we found a strong positive correlation between ENR program expression and docetaxel sensitivity of PDX models. These results strongly suggest the ENR program may be predictive of poor tumor control with AR targeting alone, *but* of improved tumor control with AR targeting + docetaxel treatment. This is an important finding because recent phase III clinical trials demonstrate that triple therapy with ADT + docetaxel + an ARPI (either abiraterone or darolutamide) is superior to ADT + docetaxel alone^64,65^. However, despite these data, many patients with hormone-naïve prostate cancer are still treated with ADT + an ARPI without docetaxel due to the lack of comparative data for triple therapy vs. ADT + an ARPI. Our results suggest that certain patients (i.e., high enza ENR program) may derive a significant benefit from adding docetaxel early on with AR-targeting.

RNA profiling data is not available from the studies of triple therapy vs. ADT + docetaxel, so we do not know whether patients with high ENR program scores are among those who derive benefit with adding an ARPI to ADT + docetaxel. Our data suggest that adding an ARPI would be unlikely to overcome the ENR program since the program we identified is linked to lower AR activity and stemness and derived from patients with enza ENR. However, it is possible that ARPIs in triplet therapy (ADT, docetaxel, and an ARPI) work by targeting AR-driven CRPC subclones that emerge with ADT + docetaxel alone.

Several kinases that regulate the cell cycle (e.g., PLK1, CDK1, CDK2, AURKA, and AURKB) were also predicted to have increased activity in ENR patients. AURKA is known to promote NEPC cell survival^45^, and the AURKA inhibitor alectinib has been tested in a clinical trial of patients with aggressive variants of prostate cancer, including those with NEPC^66^. Although that study did not meet its primary endpoint, exceptional responders were identified—namely those with overactivity of N-Myc and AURKA that interact and cooperate. We focused on CDK2 and determined that *CDK2* knockdown reduced growth of ENR-high cell lines. Our RNA-seq studies demonstrated that *CDK2* knockdown also reduced expression of the ENR program and master regulators implicated in ENR program-high tumors. Importantly, MYBL2 was among these master regulators, and a recent report demonstrates MYBL2-expressing, stem-like prostate tumors may be susceptible to CDK2 inhibition^67^.

Pharmacologic CDK2 inhibition with INX-315 recapitulated the effects of *CDK2* RNAi in our studies. Importantly, CDK2 inhibition did not re-activate AR signaling or re-sensitize AR activity-low ResA cells to enza. These results suggest that it may not be necessary to combine CDK2 inhibitors with enza to effectively target enza ENR program-high tumors. INX-315 recently entered clinical trials in patients with breast cancer or those with *CCNE1* amplifications based on prior work demonstrating CDK2 cooperates with CCNE1 (NCT05735080)^34^. However, our results suggest CDK2 is also worthy of further study and drug development efforts in prostate cancer to overcome the ENR program we identified.

There are limitations to our study that are important to acknowledge. Most notably, the number of patients used to clarify determinants of enza ENR was small. However, samples of focus were taken from a well-annotated, prospective clinical trial with longitudinal follow-up. Despite our small initial sample, unsupervised clustering correctly classified ENR tumors, and we were able to identify a gene program that we independently validated on additional, independent datasets^26,27^. We look forward to examining data from additional studies, including studies of patients treated with triple therapy or ADT + ARPI in the hormone-naïve setting to determine the importance of this program. Measuring this program may help with stratification of patients upfront to therapies that target the biology of ENR early during treatment.

Our results implicate both proliferative and EMT pathways in ENR-high tumors. Prior work demonstrates that long term ADT causes EMT associated with acquisition of a stem-like phenotype and lineage plasticity^68–70^, but these EMT, stem-like cells are often not proliferative^71–73^. Thus, it is possible that the bulk RNA-seq approach we used captured distinct subpopulations of tumor cells harboring either an EMT program or a proliferative program. Unfortunately, the tissue samples from our study have been exhausted, precluding us from addressing this possibility.

A strength of our study is the use of laser capture microdissection to profile highly tumor-rich samples, allowing us to understand critical tumor-intrinsic mechanisms of enza ENR and limiting variability of signal across samples due to tumor purity issues. However, because our samples were laser capture microdissected, we are unable to determine key microenvironmental contributions to the ENR program, which is also of significant importance.

In summary, our work demonstrates that patients with enza ENR harbor a transcriptional program linked to activation of CDK2 and proliferative, EMT, and stemness pathways. Targeting the program we identified may prove valuable for achieving more durable control than AR targeting alone.

## Methods

### Cell lines

ResA cells were kindly shared by Drs. Frank Claessens and Gerhardt Attard. LNCaP (RRID:CVCL_0395), PC3 (RRID:CVCL_0035), 22Rv1 (RRID:CVCL_1045), and ResA cells were cultured as described previously^33,74^. All cell lines were validated with STR DNA fingerprinting (Genetica) and regularly tested for Mycoplasma contamination using the MycoAlert Mycoplasma Detection Kit (Lonza cat# LT07-318).

### Chemicals

INX-315 (cat# HY-162001) and enzalutamide (cat# HY-70002) were purchased from MedChemExpress and dissolved in DMSO. DMSO served as vehicle control for the drug treatment experiments.

### Cell Viability assay

For drug dose-response experiments, indicated cells were treated in biological triplicate for 72 h with a 7-point, 5-fold dilution series from 10 mM of the indicated drugs in DMSO. Cell viability was assessed using the CellTiter-Glo (CTG) 3D Cell Viability assays (Promega cat# G9683). Dose-response was normalized to the vehicle-treated growth rate and fitted with a logistic curve as previously described^75^.

### Transient *CDK2* knockdown assays

Transient knockdowns were performed using siNTC (D-001810-10-20), siCDK2 pool (L-003236-00-0005), or siCDK2 individual siRNAs (J-003236-11-0010 and J-003236-12-0010) purchased from Horizon Discovery. The siRNA oligonucleotides were transfected with Lipofectamine 3000 (Thermo Fisher Scientific Cat# L3000015) transfection reagent. Cells were harvested 96 h post transfection to measure cell growth and for protein and RNA assays as described previously^21^.

### RNA preparation and RT-qPCR

After the indicated treatments, RNA was extracted from cells using the RNeasy Plus Mini Kit (Qiagen cat# 74034) according to the manufacturer’s protocol. After RNA extraction, 1 μg RNA was reverse-transcribed into cDNA using the High-Capacity cDNA Reverse Transcription kit (Life Technologies cat# 4368814) with random hexamer primers. RT-qPCR was performed using a Quantstudio 5 thermocycler (Applied Biosystems) with the following program: 50 °C for 2 min, 95 °C for 10 min, and 40 cycles of 95 °C for 15 s dissociation, 60 °C for 1 min annealing/extension/read. 10 μL singleplex RT-qPCR reactions contained 1X TaqMan universal PCR master mix (Thermo Fisher Scientific cat# 4304437), 1X Primer and Taqman hydrolysis probe specific to the target tested (Supplementary Data 7), and 10 ng RNA-equivalent cDNA templates. *Beta Actin* was used as endogenous control. Data were analyzed with Design and Analysis Software version 1.5.2 (Thermo Fisher Scientific).

### Western blotting

Western blotting experiments were performed by running protein lysates on SDS-PAGE gels (Thermo Fisher Scientific cat# NP0335BOX) and transferring them onto PVDF membranes as described previously^74^. Blots were probed with indicated antibodies and imaged using a Chemidoc MP imaging system (Bio-Rad). Anti-CDK2 (Cell Signaling Technology, cat# 18048), anti-GAPDH (Cell Signaling Technology, cat# 2118), and anti-Actin (Sigma-Aldrich, cat# A5441) were used for protein detection by Western blotting. Raw images of all Western blots are presented in Supplementary Fig. 4.

### Data sources and processing

We used data from the clinical trial NCT02099864, titled ‘Genetic and Molecular Mechanisms in Assessing Response in Patients with Prostate Cancer Receiving Enzalutamide’ consisting of RNA-seq data, patient survival data, and corresponding clinical annotations of tumor samples^10^, which were utilized to identify the extreme non-responder gene program. Gene expression and clinical data of independent datasets used for correlative analysis and validation of the extreme non-responder gene program were collected from the following locations: The Abida et al. dataset was downloaded according to the previously published study^26^. The microarray profiling data of the 160 patients enrolled in the CHAARTED trial (NCT00309985) were collected from Gene Expression Omnibus repository (GSE201805)^27^. The clinical data and patient outcome associated with the 160 microarray samples were requested and retrieved from NCTN/NCORP Data Archive (NCT00309985)^76^. RNA-seq data from 210 mCRPC tumor samples in the WCDT cohort are available on the EGA under study numbers EGAD00001008991, EGAD00001008487, and EGAD00001009065^32^. R packages “edgeR” (version 3.42.4) and “limma” (version 3.56.2) were employed to process and normalize the RNA-seq data and microarray values, respectively^77,78^. Organoid RNA-seq data used in this article were downloaded from the Gene Expression Omnibus (https://www.ncbi.nlm.nih.gov/geo) under accession ID GEO: GSE236808^79^. The drug response nAUC data for the organoids published previously^29^ and were kindly shared by Dr. Kathleen Kelly. RNA-seq data for the panel of prostate cancer cell lines used in the ENR program validation were published previously^33,74,80^. Differential gene expression analysis output for RNA-seq data from control cell lines and *CDK2* knockdown experiments reported in this work are provided in Supplementary Data 6 and the raw data are deposited in Gene Expression Omnibus repository (GSE282801). The RNA-seq data were converted into transcripts per kilobase million (TPM), and all expression datasets were further transformed using log1p function for downstream analyses.

### Unsupervised clustering

To understand the overall transcriptional similarities and differences between ENR and LTR tumor samples, we performed unsupervised clustering using RNA-Seq data. The raw count matrix was filtered to remove low-expression genes, retaining only those genes with a raw count of ≥10 in at least three samples. The filtered count matrix was then transformed using the vst function implemented in the DESeq2 R package (version 1.40.2)^81^. The transformed values were used to compute the sample-to-sample Euclidean distance metric for hierarchical clustering using the ‘complete’ method implemented in ComplexHeatmap package (version 2.16.0)^82,83^.

### Differential gene expression and master regulator analysis

Differential gene expression (DGE) analysis between ENR and LTR samples was performed using DESeq2 (version 1.40.2)^81^. Gene expression differences were considered significant when the adjusted *p*-value was < 0.05 and the absolute fold change was ≥ 1.5. The Wald test statistic results from DESeq2 output were used as input gene list data for master regulator analysis. The master regulator analysis was conducted using the msVIPER algorithm provided in the VIPER R package (version 1.34.0)^14^, along with ledge analysis to identify genes driving the regulator. The transcription factor and kinase regulatory networks utilized in this study were curated from several databases as previously described^84^. The cluster analysis was performed using the R package “ComplexHeatmap” (version 2.16.0) to explore differentially expressed genes between the samples from the different groups^82^. The ENR gene program was generated by selecting protein-coding genes that were differentially expressed in enza ENRs compared to LTRs, with a cutoff of adjusted *p*-value < 0.05 and an absolute fold change greater than 3. DGE and master regulator analyses between cell lines with and without CDK2 knockdown were performed using the same pipeline outlined here, where gene expression differences were considered significant when the adjusted *p*-value was < 0.05 and the absolute fold change was ≥ 2.

### Gene set enrichment analysis (GSEA) and over representation analysis

GSEA software version 4.3.2 was utilized to identify gene sets significantly activated in enza ENRs compared to LTRs, utilizing the Hallmark gene sets (h.all.v2023.1.Hs.symbols)^85,86^. Gene expression count data, normalized via variance stabilizing transformation in DESeq2, were used as inputs for GSEA^81^. Differential expression was calculated using the default Signal2Noise metric in GSEA’s permutation on gene sets. Gene sets were considered activated if their FDR q-value was below 0.05. The overrepresentation analysis of the ENR program was conducted using the fgsea (version 1.26.0) R package^87^.

### Extreme non-responder gene program analysis

The identified ENR gene program was used to construct a pseudo-regulon of ENR. Specifically, 144 up-regulated and 164 down-regulated genes (Supplementary Data 4) served as positive and negative target genes of the ENR pseudo-regulon, respectively. To assess the ENR regulon activity of each sample, the VIPER algorithm provided in the VIPER R package (version 1.34.0) was utilized^14^. A natural log-transformed gene expression matrix and a regulatory network were employed as inputs for VIPER analysis. The “viper” function from the VIPER R package was utilized to calculate the regulon activities of the ENR program and other transcription factors on different datasets. The regulatory network used in the VIPER analysis was the same as described above.

### Statistical analysis

Descriptive statistics, including median and interquartile range for continuous variables, as well as frequencies and percentages for categorical variables, were employed to summarize the transcriptomic and clinical data from the datasets. Time-to-event analysis was conducted using Kaplan–Meier survival analysis, and the log-rank test was employed to compare the two survival curves. The survival curve was generated using the R package “survminer” (version 0.5.0)^88^. The prognostic ability of the ENR program on outcomes in three independent datasets was assessed using univariable (UV) and multivariable (MV) Cox regression models with Firth’s penalized method, implemented in the R package “coxphf”^89^, resulting in Hazard ratios and 95% confidence intervals^90^. We calculated the correlation between two continuous variables using Pearson’s correlation coefficient. A *p*-value threshold of <0.05 in the Pearson correlation test was used to indicate statistical significance. Statistical analyses and figure plotting were performed using “ggplot2” (version 3.5.1)^91^ and “ggpubr” (version 0.6.0)^92^ packages in R (version 4.3.0). Statistical analysis for cell line-based assays was performed using GraphPad Prism version 10. A significance level of *p* < 0.05 was considered statistically significant.

## Supplementary information

Supplementary Information

Supplementary Data1

Supplementary Data2

Supplementary Data3

Supplementary Data4

Supplementary Data5

Supplementary Data6

Supplementary Data7

## Data Availability

The source data supporting the findings of this study are provided in the paper. Differential gene expression analysis output for RNA-seq data from *CDK2* knockdown experiments reported in this work are provided in Supplementary Table 6, and the raw data are deposited in Gene Expression Omnibus repository (GSE282801). Any additional information required for reanalyzing the data can be obtained upon request from the corresponding authors.

## Code availability

This paper does not include original code. Any additional information required for reanalyzing the data can be obtained upon request from the corresponding authors.
