# Supplementary material for: Transcriptional profiling clarifies a program of enzalutamide extreme non-response in lethal prostate cancer
Source: NPJ Precis Oncol. 2025 Jul 7;9:223. doi: 10.1038/s41698-025-01002-8 (PMC12234718; doi:10.1038/s41698-025-01002-8)
Supplement: Supplementary file 1 — Supplementary Information [file 41698_2025_1002_MOESM1_ESM.pdf]

Supplementary Information

Transcriptional profiling clarifies a program of enzalutamide extreme non-response in lethal prostate cancer

Anbarasu Kumaraswamy[1,2*], Ya-Mei Hu[3,4*], Joel A. Yates[1,2*], Chao Zhang[1,2], Eva Rodansky[1,2], Dhruv Khokhani[1,2], Diana Flores[1,2], Zhi Duan[1,2], Yi Zhang[3,4], Shaadi Tabatabaei[4], Rachel Slottke[4], Shangyuan Ye[5], Primo Lara[6], Adam Foye[7,8], Charles J. Ryan[9,10], David A. Quigley[7,11,12], Jiaoti Huang[13], Rahul Aggarwal[7,8], Robert E. Reiter[14], Max S. Wicha[1], Tomasz M. Beer[4], Matthew Rettig[14,15], Martin Gleave[16,17], Christopher P. Evans[6], Owen N. Witte[18], Joshua M. Stuart[19], George V. Thomas[4], Felix Y. Feng[7,20,#], Eric J. Small[7,8], Zheng Xia[3,4], Joshi J. Alumkal[1,2,§]

[1]Department of Internal Medicine, University of Michigan, Ann Arbor, Michigan
[2]Rogel Cancer Center, University of Michigan, Ann Arbor, Michigan
[3]Department of Biomedical Engineering, Oregon Health & Science University, Portland, Oregon
[4]Knight Cancer Institute, Oregon Health & Science University, Portland, Oregon
[5]Biostatistics Shared Resource, Knight Cancer Institute, Oregon Health & Science University, Portland, Oregon
[6]University of California Davis, Davis, California
[7]Helen Diller Family Comprehensive Cancer Center, University of California San Francisco, San Francisco, California
[8]Department of Medicine, University of California San Francisco, San Francisco, California
[9]Masonic Cancer Center, University of Minnesota, Minneapolis, Minnesota
[10]Department of Medicine, Division of Hematology, Oncology and Transplantation, University of Minnesota, Minneapolis, Minnesota
[11]Department of Urology, University of California San Francisco, San Francisco, California
[12]Department of Epidemiology & Biostatistics, University of California San Francisco, San Francisco, California
[13]Duke University, Durham, North Carolina
[14]Departments of Medicine and Urology, University of California Los Angeles, Los Angeles, California
[15]Department of Medicine, VA Greater Los Angeles Healthcare System, Los Angeles, California
[16]Department of Urological Sciences, University of British Columbia, Vancouver, British Columbia, Canada
[17]Vancouver Prostate Centre, University of British Columbia, Vancouver, British Columbia, Canada
[18]Department of Microbiology, Immunology, and Molecular Genetics at the David Geffen School of Medicine, University of California Los Angeles, Los Angeles, California
[19]Genomics Institute and Department of Biomolecular Engineering, University of California Santa Cruz, Santa Cruz, California
[20]Departments of Radiation Oncology and Urology, University of California San Francisco, San Francisco, California
[#]Deceased

*Co-first author
[§]Lead contact
Correspondence: jalumkal@med.umich.edu; xiaz@ohsu.edu

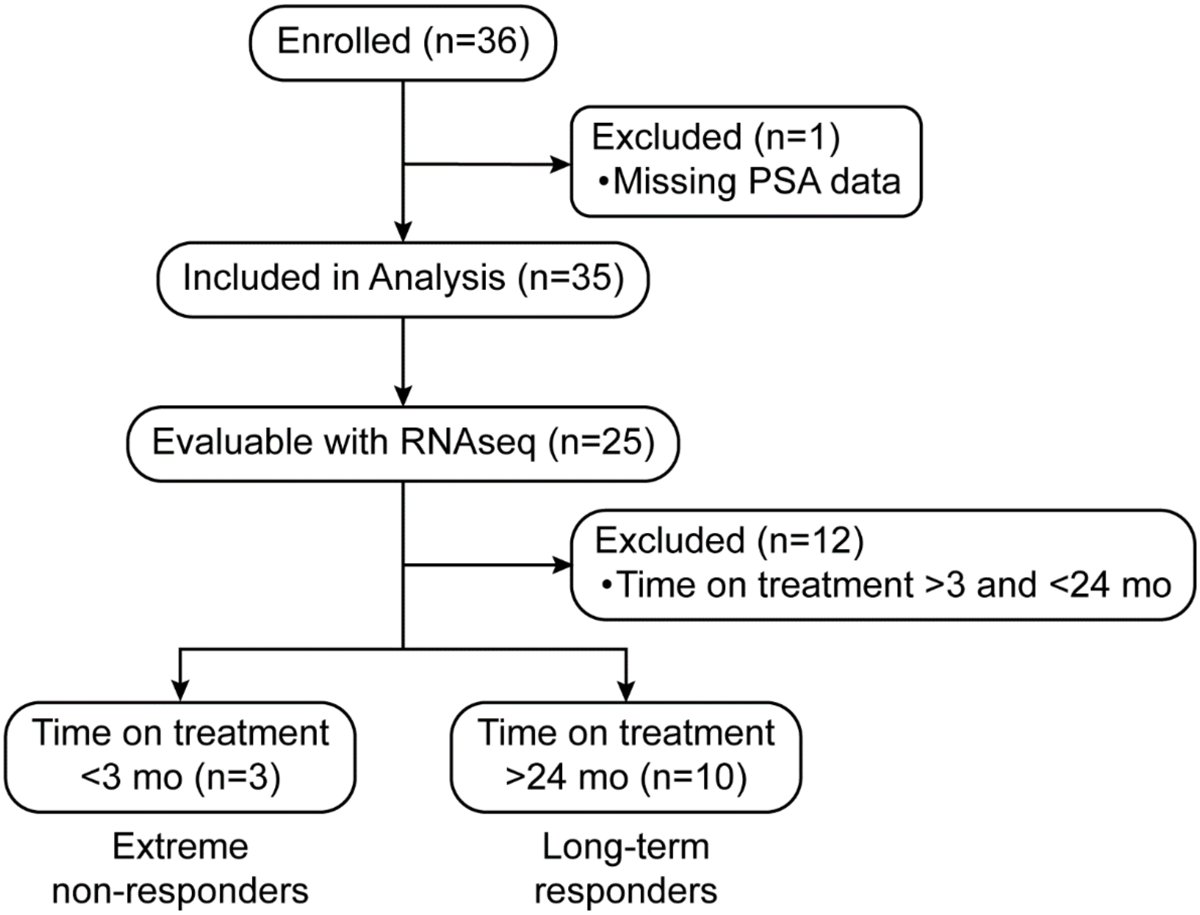

Supplementary Figure 1: Consort flow diagram

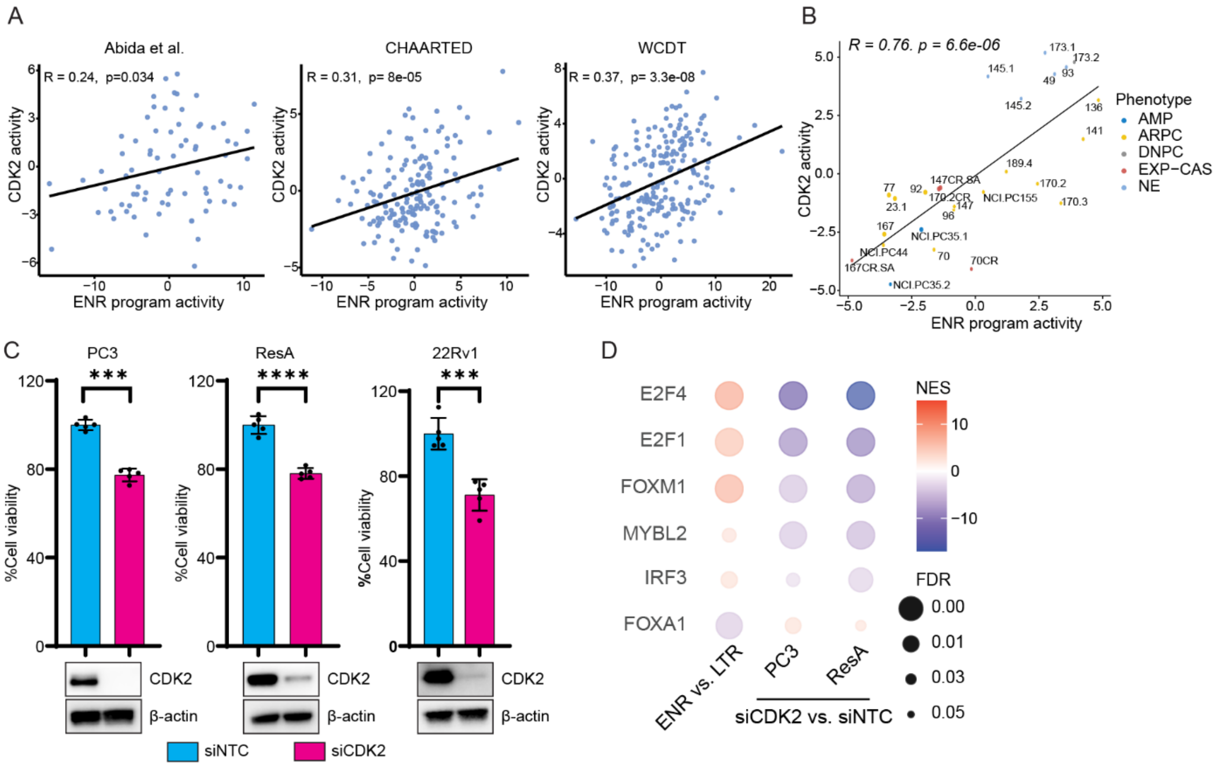

Supplementary Figure 2: CDK2 activity is correlated with expression of the extreme non-response program. A) Correlation analysis of ENR program and CDK2 activity score of patient tumors data from Abida et al.[1], CHAARTED[2], and WCDT[3] cohorts. **B)** Correlation analysis of ENR program and CDK2 activity score of patient-derived xenograft (PDX) dataset from Senatorov et al.[4]. **C)** Indicated cell lines were transfected with nontargeting control (siNTC) or *CDK2* targeting (siCDK2) siRNAs, and cell viability was measured 96 hours after transfection (top). Knockdown of *CDK2* was confirmed by Western blot analysis of cell lysates normalized to β-actin (bottom). **D)** Bubble plot depicting activity scores for indicated transcription factors from VIPER analysis of RNA seq data from ENR vs. LTR patients, or upon *CDK2* knockdown in PC3 or ResA cells.

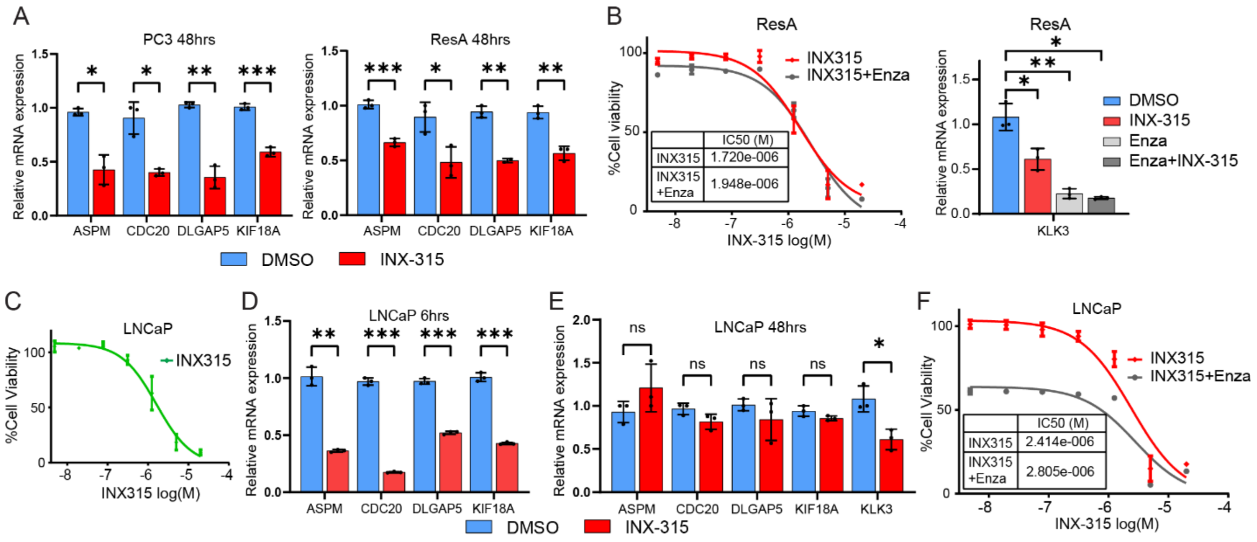

Supplementary Figure 3: **CDK2 inhibition blocks AR pathway in ENR-low model. A)** PC3 or ResA cells were treated with DMSO vehicle or 2 μM of INX-315 for 48 hrs. Expression of ENR genes were analyzed by qPCR normalized to *β-actin*. **B)** ResA cells washed out of enzalutamide for 6 days were treated with increasing doses of INX-315 with or without 10 μM enzalutamide for 72 hours to generate dose-response viability curves (Left). *KLK3* expression was measured by qPCR normalized to *β-actin* (Right). **C)** LNCaP cells were treated with increasing doses of INX-315 for 72 hours to generate dose-response viability curve. **D, E)** LNCaP cells were treated with DMSO vehicle or 2 μM of INX-315 for 6 hrs (D) or 48 hrs (E). Expression of indicated genes were analyzed by qPCR normalized to *β-actin*. **F)** LNCaP cells were treated with increasing doses of INX-315 with or without 10 μM enzalutamide for 72 hours to generate dose-response viability.

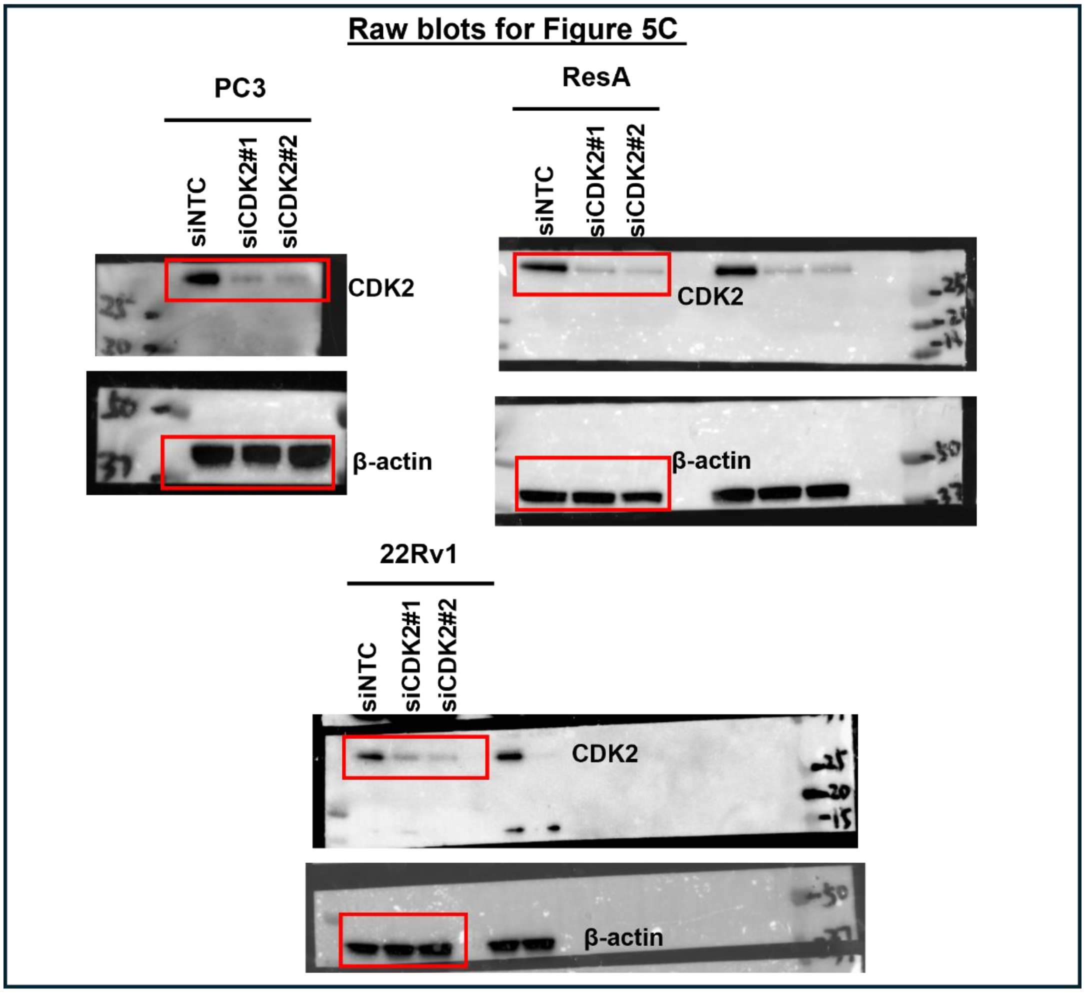

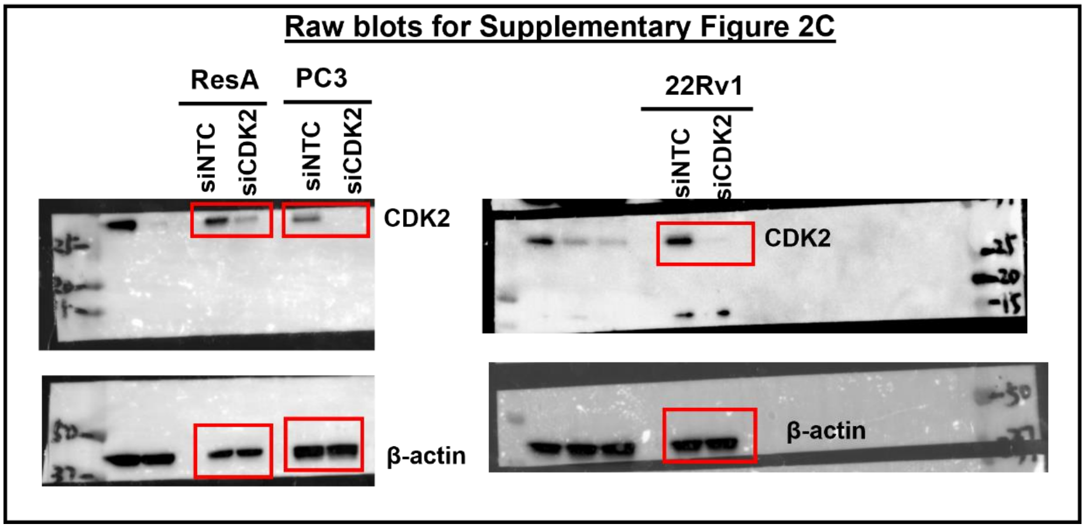

Supplementary Figure 4: Raw images for Western blots presented in Figure 5C and Supplementary Figure 2C.

Supplementary Data:

Supplementary Data 1 Patient characteristics

Supplementary Data 2 Time on treatment, overall survival, and PSA response data

Supplementary Data 3 Master regulator transcription factors and kinases in extreme non-response (ENR) vs. long-term response (LTR) patients

Supplementary Data 4 Extreme non-response (ENR) program gene list

Supplementary Data 5 Kaplan-Meier survival analysis values

Supplementary Data 6 Differentially-expressed genes from siCDK2 in PC3 and ResA

Supplementary Data 7 Taqman qPCR assays

Supplementary References:

1. Abida W, Cyrta J, Heller G, et al: Genomic correlates of clinical outcome in advanced prostate cancer. Proc Natl Acad Sci U S A 116:11428-11436, 2019
2. Hamid AA, Huang HC, Wang V, et al: Transcriptional profiling of primary prostate tumor in metastatic hormone-sensitive prostate cancer and association with clinical outcomes: correlative analysis of the E3805 CHAARTED trial. Ann Oncol 32:1157-1166, 2021
3. Lundberg A, Zhang M, Aggarwal R, et al: The Genomic and Epigenomic Landscape of Double-Negative Metastatic Prostate Cancer. Cancer Res 83:2763-2774, 2023
4. Senatorov IS, Bowman J, Jansson KH, et al: Castrate-resistant prostate cancer response to taxane is determined by an HNF1-dependent apoptosis resistance circuit. Cell Rep Med 5:101868, 2024
